# Patients’ Satisfaction With Telepsychiatry Services at a University Hospital in Riyadh During the COVID-19 Pandemic

**DOI:** 10.7759/cureus.17307

**Published:** 2021-08-19

**Authors:** Ahmad M Almalky, Fatima A Alhaidar

**Affiliations:** 1 Faculty of Medicine, Jazan University, Jazan, SAU; 2 Faculty of Medicine, King Saud University, Riyadh, SAU; 3 Child and Adolescent Psychiatric Department, King Khalid University Hospital, Riyadh, SAU

**Keywords:** telepsychiatry, satisfaction, patients' perspectives, telemedicine, mental health, pandemic

## Abstract

Background and objective

Telepsychiatry uses electronic communication and various technologies to provide psychiatric care by a psychiatrist in one location to a patient in another location. It was originally created to meet the mental health needs of patients in rural, remote, and inaccessible areas. This study aimed to assess the satisfaction level with telepsychiatry from patients' perspectives and to study whether the satisfaction levels influence the patients' decision to use the service in the future.

Methodology

This was a cross-sectional survey study conducted at King Khalid University Hospital in Riyadh, Saudi Arabia. The study included psychiatric patients with complete medical records who were followed up through the telepsychiatry program over the phone due to the restriction and regulation implemented by the government in the wake of the coronavirus disease 2019 (COVID-19) pandemic. The data were collected via a questionnaire designed on a Google Form. Initially, the sample size was set at 337 psychiatric patients, but only 141 patients agreed to be included. The SPSS Statistics program (IBM, Armonk, NY) was used to analyze the data.

Results

Patients were generally satisfied with the telepsychiatry services; 80.1%, 95.7%, and 96.5% of the participants were satisfied with the structure, process, and outcome, respectively, and 94.3% of the patients reported a sense of overall satisfaction. The study did not find any relationship between satisfaction and demographic characteristics. Patients highly valued some aspects during the service use, such as comfort, privacy, easy access, carefulness, and skillfulness of the clinicians. Of the respondents, 24.1% agreed and 24.8% strongly agreed when asked if they would use the service in the future.

Conclusion

Patients had generally positive satisfaction levels toward telepsychiatry service, and many reported that they would like to continue using it in the future. However, further studies are needed to assess whether patient perception will change over time after the COVID-19 pandemic.

## Introduction

Telepsychiatry involves the use of electronic communication and various technologies to provide psychiatric care by a psychiatrist in one location to a patient in a different location virtually, either through voice or video sessions [1]. Telepsychiatry was originally created to meet the mental health needs of patients in rural, remote, and inaccessible areas [2,3]. Moreover, evidence shows that the use of telepsychiatry has helped to overcome many previous issues including shortages of mental health clinicians [4], social stigma attributed to treatment in a mental health setting, as well as in the context of a pandemic such as the one caused by coronavirus disease 2019 (COVID-19) [5-7]. As the use of telepsychiatry in healthcare has dramatically increased in the past decade, it has attracted significant attention from researchers and psychiatrists [8]. In light of this, it has become an element of key importance to have a proper understanding as to how satisfied the patients actually are with the service they receive from the telepsychiatry approach [8,9]. Furthermore, a rapidly growing number of studies in several medical fields have observed that patients’ attitudes play a critical role in healthcare outcomes [10,11]. This highlights the need for more research to understand patients' satisfaction levels with the telepsychiatry service.

In general, published studies about telepsychiatry are limited and consist of small sample sizes [12,13]. In addition, the current level of evidence is insufficient to provide generalizable results [14,15]. And, the current level of evidence regarding patients' satisfaction with the use of telepsychiatry in Saudi Arabia remains very limited. Therefore, we conducted this cross-sectional study to assess the satisfaction level with telepsychiatry from patients' perspectives and to examine whether satisfaction levels affect the patients' well-being.

## Materials and methods

This was a cross-sectional survey conducted at King Khalid University Hospital in Riyadh, Saudi Arabia. The study included a cohort of psychiatric patients with complete medical records who were followed up through the telepsychiatry program over the phone owing to the restrictions and curfew implemented by the government at King Khalid University Hospital due to the ongoing COVID-19 pandemic. This study involved the distribution of online questionnaires on Google Forms, which were designed by the researcher and piloted before the actual data collection.

The Telepsychiatry Care Satisfaction Questionnaire (TCSQ) was formulated from two previously validated surveys [16,17]. First, the Psychiatric Care Satisfaction Questionnaire (PCSQ), which was validated and used in previous research [16]. Secondly, the Telehealth Satisfaction Scale (TeSS), which is a 10-question scale survey that was used in a study research regarding telehealth satisfaction [17]. The TCSQ questionnaire was reconstructed to assess the views of psychiatric patients about the healthcare providers that are directly involved in their treatment plan. These views included the structure of the program, the process that was carried out, and its outcomes. This questionnaire comprised 26 items in total, of which seven items were used for collecting demographic data. The TCSQ employs a five-point Likert scale (strongly agree, agree, uncertain, disagree, and strongly disagree). Each question was given a score ranging from 1 to 5; higher scores imply satisfaction with that questionnaire item. The TCSQ statements are both negative and positive, direct, and indirect. Feedback from three different psychiatrists was taken to further validate the questionnaire. Furthermore, in order to validate the language part of the questionnaire, it was translated into Arabic and back to English by an independent translator. Afterward, the translated questionnaire was translated back to English and was checked for accuracy because of the possibility that some rephrasing might be lost in translation due to the sophistication of the Arabic language.

All individuals who were enrolled in the telepsychiatry program were contacted and informed about the process of the current survey-based study. All patients who gave informed consent to be part of the study were included. The participants were contacted by phone to explain the study objectives, and then a link to the questionnaire was sent to them by Whatsapp to answer the survey. The inclusion criteria were as follows: documented psychiatry cases who were followed up at King Khalid University Hospital, patients with full medical records, patients who were compliant with the assigned treatment plans and sessions, and patients who were willing to take part in our survey-based study. On the other hand, new psychiatric cases, patients who did not follow up, patients with incomplete medical records, and patients who were unwilling to participate in our survey study were excluded. There was no restriction on age or medical conditions as the guardians were asked to fill in the survey on behalf of the patients. Written consents were collected by getting the participants to check a box on the Google Form link, which had information about the study objective and assurance of complete confidentiality of the provided data as well as a guarantee to not collect personal information.

Initially, a pilot study was conducted to assess the validity and reliability of the developed questionnaire among 10% of the overall sample through a simple randomization process. Cronbach's alpha coefficient was utilized to find the reliability for each instrument. The alpha coefficient was high for the instruments with a value of 0.832. Following the validation of the questionnaire, we asked all included participants to fill in the online questionnaire. Individuals who were not able to fill the online questionnaire were asked to complete the study surveys through a telephone-based interview. The study was approved by the Institutional Review Board (IRB)-Ethics Committee of King Khalid University Hospital. Furthermore, those who were 18 years and younger or mentally impaired to provide consent on their own had their questionnaires filled in with the help of their guardians/parents and the consent was obtained from their legal guardians. The youngest patient included in the study was six years old and the oldest was 85 years old. The researcher used the SPSS Statistics program version 25 (IBM, Armonk, NY) for data entry and analysis. The first stage of data entry involved constructing the entry base and coding of variables, followed by actual data entry. At the analysis stage, data cleaning and data management for the variables of interest were performed. A five-point Likert scale was used, and the points awarded ranged from 1 point for "strongly disagreeing" to 5 points for "strongly agreeing." A reverse coding was done for the reverse statements. A collective score was used to calculate the satisfaction score by the summation of all statements. The middle point was considered as the cut-off point between the "satisfied" and "not satisfied" categories. Descriptive analysis including figures and frequency tables were used to describe the main features of the data. The Chi-square test was used to examine the relationship between satisfaction categories and other variables. The confidence interval was considered at 95%, and a p-value <0.05 was considered statistically significant.

## Results

Table 1 shows the demographic characteristics of patients. A total of 141 participants were included in the study. Out of those, 56% were females, 95.7% were Saudi, 43.3% had a bachelor’s degree, 85.8% resided in Riyadh, 61% were unemployed, and 48.2% were married. The mean age of the participants was 39.39 years.

**Table 1:** Participants' demographic characteristics

Variables	Categories	N (%)
Gender	Male	62 (44%)
	Female	79 (56%)
Nationality	Saudi	135 (95.7%)
	Non-Saudi	6 (4.3%)
Educational qualification	Illiterate	7 (5%)
	Special needs	6 (4.3%)
	Primary school	11 (7.8%)
	Middle school	7 (5%)
	High school	41 (29.1%)
	Diploma	4 (2.8%)
	Bachelor’s degree	61 (43.3%)
	Master’s degree	3 (2.1%)
	PhD	1 (0.7%)
Place of residence	Riyadh	121 (85.8%)
	Outside Riyadh	20 (14.2%)
Employment status	Employed	51 (36.2%)
	Unemployed	86 (61%)
	Self-employed	4 (2.8%)
Marital status	Single	60 (42.6%)
	Married	68 (48.2%)
	Widowed	5 (3.5%)
	Divorced	8 (5.7%)

Regarding the response on the telepsychiatry service, about 65.3% of patients were satisfied and strongly satisfied with the duration of the visits, 63.4% were comfortable using the telepsychiatry service, 56.7% of patients were at ease about reaching the telepsychiatry department, 52.1% were satisfied with how the department acted in case the patient was out of reach, and only 32.2% thought that doctors needed more training to conduct a voice session. About 46.8% and 38.3% of the patients were satisfied and strongly satisfied that the psychiatrist gave them enough space to listen to their problems and concerns, respectively. Moreover, 42.6% were satisfied and 36.2% were strongly satisfied that the psychiatrist explained the treatment plan well. Also, 37.6% were satisfied and 36.2% were strongly satisfied with the quality of the audio communication. Almost half of the respondents (42.6%) were satisfied and 34% were strongly satisfied with the thoroughness, carefulness, and skillfulness of the clinicians. In addition, less than a third of the respondents (24.1%) were satisfied and 27% were strongly satisfied regarding the ease in discussing matters with a call rather than an outpatient appointment. In contrast, 30.5% were dissatisfied and 43.3% were strongly dissatisfied with the use of technical terms that patients do not understand. Regarding the privacy of the care, 35.5% of patients agreed and almost half of them (48.2%) strongly agreed that it was satisfactory, while 38.3% agreed and 33.3% strongly agreed that using telepsychiatry service will help more patients in the future (Table 2). Also, 39% and 27% of the patients agreed and strongly agreed, respectively, that they were satisfied with the rate of follow-up they received from the telepsychiatry team. Moreover, 41.1% agreed and 20.6% strongly agreed with the view that there are more things regarding telepsychiatry with regard to the case that can be improved (Table 2). Around 28% agreed that it was easy to receive their prescription and pills using the online prescription tools while 32.6% of the patients strongly agreed with the same statement. On the ease of visiting the hospital for laboratory work on an appointment basis, 28.4% agreed and 32.6% strongly agreed on that fact. Lastly, a low percentage of patients (15.6%) agreed that it was difficult to see the doctor in person when needed while 28.4% strongly agreed on that matter (Table 2).

**Table 2:** Patients’ responses to the telepsychiatry questionnaire SD: strongly disagree; D: disagree; N: neutral; A: agree; SA: strongly agree

Statements	SD	D	N	A	SA	Mean
Patients’ responses to the service structure statements						
I am satisfied with the duration of the session	14 (9.9%)	13 (9.2%)	22 (15.6%)	50 (35.5%)	42 (29.8%)	3.7
I am comfortable in using telepsychiatry service	19 (13.5%)	19 (13.5%)	15 (10.6%)	48 (34%)	40 (28.4%)	3.5
I am satisfied with the ease of reaching the telepsychiatry department	19 (13.5%)	16 (11.3%)	26 (18.4%)	44 (31.2%)	36 (25.5%)	3.4
I am satisfied with how the department act in case I am out of reach	19 (13.5%)	18 (12.8%)	30 (21.3%)	53 (37.6%)	21 (14.9%)	3.3
I think the doctors need more training to conduct a voice session	12 (8.5%)	38 (27%)	48 (34%)	20 (14.2%)	23 (16.3%)	3.0
Patients’ responses to the service process statements						
The psychiatrist gives you space to listen to your problems and concerns	3 (2.1%)	4 (2.8%)	14 (9.9%)	66 (46.8%)	54 (38.3%)	4.16
The psychiatrist explains the treatment plan well enough	1 (0.7%)	7 (5%)	22 (15.6%)	60 (42.6%)	51 (36.2%)	4.09
I am satisfied with the audio quality in communication	4 (2.8%)	5 (3.5%)	28 (19.9%)	53 (37.6%)	51 (36.2%)	4.01
I am satisfied with the thoroughness, carefulness, and skillfulness of the clinicians	6 (4.3%)	3 (2.1%)	24 (17%)	60 (42.6%)	48 (34%)	4.00
I find it easier to discuss matters during a call rather than an outpatient appointment	18 (12.8%)	24 (17%)	27 (19.1%)	34 (24.1%)	38 (27%)	3.35
The doctor uses technical terms that you don’t understand	43 (30.5%)	61 (43.3%)	22 (15.6%)	13 (9.2%)	2 (1.4%)	2.08
Patients’ responses to the service outcome statements						
I am satisfied with the privacy level of my case	3 (2.1%)	3 (2.1%)	17 (12.1%)	50 (35.5%)	68 (48.2%)	4.3
I think telepsychiatry will help reach more patients in the future	4 (2.8%)	14 (9.9%)	22 (15.6%)	54 (38.3%)	47 (33.3%)	3.9
I am satisfied with the rate of follow-up I receive from the telepsychiatry department	9 (6.4%)	17 (12.1%)	21 (14.9%)	55 (39%)	39 (27.7%)	3.7
I think there are more things regarding telepsychiatry with regard to my case that can be improved	3 (2.1%)	12 (8.5%)	39 (27.7%)	58 (41.1%)	29 (20.6%)	3.7
I find it easy to receive my prescription and pills using online prescriptions	15 (10.6%)	16 (11.3%)	24 (17%)	40 (28.4%)	46 (32.6%)	3.6
I find it easy to visit the hospital for laboratory work on an appointment basis	15 (10.6%)	13 (9.2%)	21 (14.9%)	52 (36.9%)	40 (28.4%)	3.6
It is hard to see a doctor face-to-face when I need to	14 (9.9%)	35 (24.8%)	30 (21.3%)	22 (15.6%)	40 (28.4%)	3.3

On further analysis, there was no statistically significant relationship between patients' satisfaction with the service structure and patients' demographic characteristics (Table 3). Furthermore, there was no statistically significant relationship between patients' satisfaction with the service process and patients' demographic characteristics (Table 4), and there was no statistically significant relationship between patients' satisfaction with the service outcome and patients' demographics (Table 5). Similarly, there was no statistically significant relationship between patients' general satisfaction with the service and patients' demographic characteristics (Table 6).

**Table 3:** Relationship between patients’ satisfaction with the service structure and demographic characteristics

Variables	Dissatisfied	Satisfied	P-value
Gender			
Male	8 (12.9%)	54 (87.1%)	0.051
Female	20 (25.3%)	59 (74.7%)	
Age			
<=18 years	2 (20.0%)	8 (80.0%)	0.215
19-30 years	8 (33.3%)	16 (66.7%)	
31-50 years	11 (14.3%)	66 (85.7%)	
>=50 years	7 (23.3%)	23 (76.7%)	
Nationality			
Saudi	27 (20.0%)	108 (80.0%)	0.659
Non-Saudi	1 (16.7%)	5 (83.3%)	
Educational qualification			
Higher school or less	13 (18.1%)	59 (81.9%)	0.848
University education	15 (23.1%)	50 (76.9%)	
Postgraduate	0 (0%)	4 (100.0%)	
Place of residence			
Riyadh	22 (18.2%)	99 (81.8%)	0.175
Outside Riyadh	6 (30.0%)	14 (70.0%)	
Employment status			
Employed	8 (15.7%)	43 (84.3%)	0.184
Unemployed	18 (20.9%)	68 (79.1%)	
Self-employed	2 (50.0%)	2 (50.0%)	
Marital status			
Single	13 (21.7%)	47 (78.3%)	0.582
Married	13 (19.1%)	55 (80.9%)	
Other	2 (15.4%)	11 (84.6%)	

**Table 4:** Relationship between patients’ satisfaction with the service process and demographic characteristics

Variables	Dissatisfied	Satisfied	P-value
Gender			
Male	1 (1.6%)	61 (98.4%)	0.171
Female	5 (6.3%)	74 (93.7%)	
Age			
<=18 years	0 (0%)	10 (100%)	0.121
19-30 years	3 (12.5%)	21 (87.5%)	
31-50 years	3 (3.9%)	74 (96.1%)	
>=50 years	0 (0%)	30 (100%)	
Nationality			
Saudi	6 (4.4%)	129 (95.6%)	0.767
Non-Saudi	0 (0%)	6 (100%)	
Educational qualification			
Higher school or less	2 (2.8%)	70 (97.2%)	0.502
University education	4 (6.2%)	61 (93.8%)	
Postgraduate	0 (0%)	4 (100%)	
Place of residence			
Riyadh	6 (5%)	115 (95%)	0.311
Outside Riyadh	0 (0%)	20 (100%)	
Employment status			
Employed	2 (3.9%)	49 (96.1%)	0.431
Unemployed	3 (3.5%)	83 (96.5%)	
Self-employed	1 (25%)	3 (75%)	
Marital status			
Single	3 (5%)	57 (95%)	0.718
Married	3 (4.4%)	65 (95.6%)	
Other	0 (0%)	13 (100%)	

**Table 5:** Relationship between patients’ satisfaction with service outcomes and demographic characteristics

Variables	Dissatisfied	Satisfied	P-value
Gender			
Male	0 (0%)	62 (100%)	0.052
Female	5 (6.3%)	74 (93.7%)	
Age			
<=18 years	0 (0%)	10 (100%)	0.063
19-30 years	3 (12.5%)	21 (87.5%)	
31-50 years	2 (2.6%)	75 (97.4%)	
>=50 years	0 (0%)	30 (100%)	
Nationality			
Saudi	5 (3.7%)	130 (96.3%)	0.632
Non-Saudi	0 (0%)	6 (100%)	
Educational qualification			
Higher school or less	1 (1.4%)	71 (98.6%)	0.247
University education	4 (6.2%)	61 (93.8%)	
Postgraduate	0 (0%)	4 (100%)	
Place of residence			
Riyadh	5 (4.1%)	116 (95.9%)	0.355
Outside Riyadh	0 (0%)	20 (100%)	
Employment status			
Employed	1 (2%)	50 (98%)	0.056
Unemployed	3 (3.5%)	83 (96.5%)	
Self-employed	1 (25%)	3 (75%)	
Marital status			
Single	3 (5%)	57 (95%)	0.631
Married	2 (2.9%)	66 (97.1%)	

**Table 6:** Relationship between patients' general satisfaction about the service and demographic characteristics

Variables	Dissatisfied	Satisfied	P-value
Gender			
Male	3 (4.8%)	59 (95.2%)	0.500
Female	5 (6.3%)	74 (93.7%)	
Age			
<=18 years	0 (0%)	10 (100%)	0.362
19-30 years	3 (12.5%)	21 (87.5%)	
31-50 years	3 (3.9%)	74 (96.1%)	
>=50 years	2 (6.7%)	28 (93.3%)	
Nationality			
Saudi	8 (5.9%)	127 (94.1%)	0.700
Non-Saudi	0 (0%)	6 (100%)	
Educational qualification			
Higher school or less	2 (2.8%)	70 (97.2%)	0.234
University education	6 (9.2%)	59 (90.8%)	
Postgraduate	0 (0%)	4 (100%)	
Place of residence			
Riyadh	8 (6.6%)	113 (93.4%)	0.236
Outside Riyadh	0 (0%)	20 (100%)	
Employment status			
Employed	4 (7.8%)	47 (92.2%)	0.135
Unemployed	3 (3.5%)	83 (96.5%)	
Self-employed	1 (25%)	3 (75%)	
Marital status			
Single	3 (5%)	57 (95%)	0.551
Married	5 (7.4%)	63 (92.6%)	
Others	0 (0%)	13 (100%)	

However, regarding the relationship between satisfaction categories and the views of the patients about their desire to continue using the telepsychiatry service in the future, statistically significant differences were found in satisfaction levels with respect to all items (structure, process, outcome, and general satisfaction), as well as the patients’ preference to use the service in the future, as all p-values were less than 0.05 (Table 7).

**Table 7:** Relationship between patients' general satisfaction with the service and their willingness to continue with the telepsychiatry service *Statistically significant SD: strongly disagree; D: disagree; N: neutral; A: agree; SA: strongly agree

Variables	SD	D	N	A	SA	P-value
Satisfaction with the structure of the service						
Dissatisfied	14 (50%)	10 (35.7%)	0 (0%)	1 (3.3%)	3 (10.7%)	0.000*
Satisfied	3 (2.7%)	22 (19.5%)	23 (20.4%)	33 (29.2%)	32 (28.3%)	
Satisfaction with the process of the service						
Dissatisfied	4 (66.7%)	2 (33.3%)	0 (0%)	0 (0%)	0 (0%)	0.001*
Satisfied	13 (9.6%)	30 (22.2%)	23 (17%)	34 (25.2%)	35 (25.9%)	
Satisfaction with the outcome of the service						
Dissatisfied	4 (80%)	1 (20%)	0 (0%)	0 (0%)	0 (0%)	0.000*
Satisfied	13 (9.6%)	31 (22.8%)	23 (16.9%)	34 (25%)	35 (25.7%)	
General satisfaction with the service						
Dissatisfied	4 (50%)	2 (25%)	0 (0%)	0 (0%)	2 (25%)	0.009*
Satisfied	13 (9.8%)	30 (22.6%)	23 (17.3%)	34 (25.6%)	33 (24.8%)	

## Discussion

Among the patients who participated in the telepsychiatry service, the majority (94.3%) were satisfied with the quality of the healthcare they received. The patients' satisfaction was in line with the existing literature that found patients' satisfaction with the care quality and clinical outcomes following telehealth visits superior to those of traditional medical setting visits [18-20]. Our study did not find any differences in satisfaction levels with respect to patients’ demographic characteristics. This result is consistent with previous related studies that failed to show differences in patients’ satisfaction scores pertaining to their demographics. However, lack of medical insurance was a significant predictor for previous preferences for telehealth service [19], while the distance from patients’ homes was not measured in the current study due to the pandemic rules. The study revealed that patients were satisfied with the duration of the session they received, comfortability, ease of reaching to the telepsychiatry setting, quality, carefulness, appointment, and privacy. Consistent with our findings, a previous related study has shown the satisfaction level of patients with telepsychiatry services to be positive [21]. Another study showed that 63.6% of patients either agreed or strongly agreed that remote treatment sessions (telephone or video) have been just as helpful as in-person sessions [22]. Also, it showed good satisfaction levels among the psychiatric patients with the appointment confidentiality, privacy, and comfortability of telepsychiatry services. In addition, it showed that improved access to care increases the patients' satisfaction with the service [23].

About half of the participants either agreed or strongly agreed to continue using the telepsychiatry service in the future, and patients’ satisfaction score was associated with their willingness to use the service in the future. In a similar study, 64.2% of the participants either agreed or strongly agreed with the statement that they would consider using telepsychiatry sessions in the future [22]. Hence, this perception may be driven by certain specific aspects, which may include limited closeness to the medical staff, lack of ability to note the body language, nonverbal cues, and physical signs of the disease [24]. However, movement restrictions associated with the COVID-19 pandemic have made the medical setting visits less interesting to the patients, which may overestimate the real satisfaction scores. The study showed a statistically significant relationship between patients' satisfaction levels and patients' preference to continue using the service in the future.

Limitations of the study include a relatively small sample size that was not representative of the general population. Furthermore, the online sessions were done during lockdown periods, and hence the respondents may have a biased opinion about normalizing the process given the ongoing pandemic. However, longitudinal studies should be conducted to validate these results once the COVID-19 pandemic ends.

## Conclusions

A large segment of the patient population was satisfied with the telephonic consultative services. Patients considered privacy, confidentiality, comfortability, ease of reach to the service, appointment, and carefulness of the healthcare providers to be the best attributes regarding the telepsychiatry service, with about half of the participants expressing the desire to use it in the future. However, further research should be conducted in order to confirm and validate these findings once the COVID-19 pandemic ends.
